# Supplementary material for: Cell-free biogenesis of bacterial division proto-rings that can constrict liposomes
Source: Commun Biol. 2020 Sep 30;3:539. doi: 10.1038/s42003-020-01258-9 (PMC7527988; doi:10.1038/s42003-020-01258-9)
Supplement: Supplementary file 2 — Description of additional supplementary files [file 42003_2020_1258_MOESM2_ESM.docx]

Description of Additional Supplementary Files

Supplementary Data 1: Source data underlying plots shown in figures.

Supplementary Movie 1: Ring-like structures imaged with TIRF and SIM microscopy showing that FtsA-FtsZ bundles on SLB are dynamic. Movies of the FtsZ-A647 fluorescence signal are displayed on the left and the corresponding kymographs are shown on the right. Sample preparation was as described in main text Fig. 1a,b and in Supplementary Fig. 1.

Supplementary Movie 2: Time series of confocal fluorescence images showing that in-liposome synthesized FtsA assembles with FtsZ into ring-like structures that drive membrane neck formation and vesicle budding. Ring-forming protein clusters localized at a constriction site can split, which induces multiple necks separated by blebbing vesicles. The ftsAopt DNA template was expressed within phospholipid vesicles in the presence of 3 µM purified FtsZ-A647. Green signal, membrane dye fluorescence; magenta signal, FtsZ-A647 fluorescence. Scale bar represents 10 µm.
